# Influential Factors of an Asynchronous BCI for Movement Intention Detection

**DOI:** 10.1155/2020/8573754

**Published:** 2020-03-23

**Authors:** Sura Rodpongpun, Thapanan Janyalikit, Chotirat Ann Ratanamahatana

**Affiliations:** Department of Computer Engineering, Chulalongkorn University, Pathumwan, Bangkok 10330, Thailand

## Abstract

In recent years, asynchronous brain computer interface (BCI) systems have been utilized in many domains such as robot controlling, assistive technology, and rehabilitation. In such BCI systems, movement intention detection algorithms are used to detect movement desires. In recent years, movement-related cortical potential (MRCP), an electroencephalogram (EEG) pattern representing voluntary movement intention, attracts wide attention in movement intention detection. Unfortunately, low MRCP detection accuracy makes the asynchronous BCI system impractical for real usage. In order to develop an effective MRCP detection algorithm, EEG data have to be properly preprocessed. In this work, we investigate the relationship and effects of three factors including frequency bands, spatial filters, and classifiers on MRCP classification performance to determine best settings. In particular, we performed a systematic performance investigation on combinations of five frequency bands, five spatial filters, and six classifiers. The EEG data were acquired from subjects performing series of self-paced ankle dorsiflexions. Analysis of variance (ANOVA) statistical test was performed on F1 scores to investigate effects of these three factors. The results show that frequency bands and spatial filters depend on each other. The combinations directly affect the F1 scores, so they have to be chosen carefully. The results can be used as guidelines for BCI researchers to effectively design a preprocessing method for an advanced asynchronous BCI system, which can assist the stroke rehabilitation.

## 1. Introduction

A brain computer interface (BCI) system is a system that translates human minds to control signals for external devices. These external devices include simple feedback systems and more complex devices such as prosthetic organs [1]. Moreover, a BCI system can be used for communication between persons. It transmits one's messages as brain activity signals to other persons through a system that can decode the signals to human recognizable messages. In this sense, patients suffering from a brain disease including locked-in syndrome (LIS) or completely locked-in state (CLIS), as they cannot move, talk, or even blink, can get benefits from the BCI system [2]. Therefore, large amount of research effort has been devoted to solving communication problem for these patients [1, 3–5].

In the past decade, there has been evidence based on the Hebbian theory [6] showing that motor function loss can be restored by brain plasticity induction through the BCI system for rehabilitation [7]. This phenomenon can be a great advantage for stroke patients, whose brain regions were partially destroyed by blood clots in the brain vessel. This situation is a cause of mobility and speech function impairment that affects the patients' daily life. After some treatments and a long-term rehabilitation program, some patients can partially or even fully recover their motor functions; it depends on how long the symptom lasts, how much the brain region is destroyed, how well the brain plasticity develops, and even how good the rehabilitation program is.

BCI systems can be categorized by their functions into two types: synchronous and asynchronous. For the synchronous BCI system, aka a cue-based BCI system, users have to perform specific task in response to given cues while using the system. This scenario often causes discomfort and fatigue. For this reason, asynchronous BCI systems have been proposed since the last decade [1]. The asynchronous BCI system, aka a self-paced BCI system, allows its users to perform tasks upon their desire. In this sense, the asynchronous BCI system overcomes the ordinary synchronous BCI system by providing more comfort and causing less fatigue. These properties are very important to the BCI system for rehabilitation because the users have to interact with the system for a long period. However, designing an asynchronous BCI system is much more challenging due to the fact that the acquired signals are noisier because users can pay less concentration to use the system.

Recently, the asynchronous BCI system has been used in stroke rehabilitation for the first time to induce brain plasticity [8]. The asynchronous BCI system is applied in stroke rehabilitation by using brain signals measured from the scalp, known as electroencephalogram (EEG), to drive a rehabilitation tool. Particularly, movement intention is detected from EEG data and translated to a control signal to instantaneously switch on an electrical stimulator. Upon completion of some rehabilitation sessions, stroke impact scale (SIS) appears to improve in all patients, demonstrating that impacts on stoke patients could be alleviated. The key of these improvements is the preciseness of the electrical stimulator activation when patients start to move or start to imagine about moving the affected limb [9]. Although there have been evidences showing that the recent BCI systems for stroke rehabilitation can induce brain plasticity in stroke patients [8–10], its limitation in terms of movement intention detection accuracy makes it not widely used.

To detect the movement intention, there are two types of signals that are usually utilized: movement-related cortical potential (MRCP) [11] and event-related desynchronization/synchronization (ERD/ERS). These two come from different domains; MRCP is extracted from a time domain as a pattern of time series signal while ERD/ERS is extracted from a frequency domain. In terms of movement intention detection accuracy, MRCP and ERD/ERS provide comparable detection accuracy. However, it has been recently shown that MRCP is superior in terms of detection latency and therefore is preferred to ERD/ERS for movement intention detection [12, 13].

Over the past decade, many algorithms have been proposed to detect the movement intention before real movement execution [14–18]; however, it is still unclear about how to filter or clean the acquired signals before feeding them to a classifier. For example, Niazi et al. [16] and Lin et al. [17] proposed methods utilizing matched filter (MF) and locality sensitivity discriminant analysis (LSDA) to detect movement intentions from MRCP, but they suffered from difficult choices in selecting both an appropriate frequency band and a spatial filtering technique. Instead of finding an appropriate solution, the choices were made arbitrarily.

Although some researchers were aware of these problems and attempted to explore an appropriate configuration or combination of computation methods for the movement intention or the movement imagination detection in BCI system [14, 19–24], none of them explored these problems thoroughly. The most systematic study was in [14] where combinations among three factors of spatial filter, temporal filter, and classifier were analyzed. However, the work only focuses on movement intention classification between left and right hand, not on movement intention detection. Moreover, not only MRCP or EEG data in time domain but also ERD/ERS data were used as it can be an obstacle for a real-time usage. In [24], the authors studied the effects of frequency and spatial filters on the contingent negative variation (CNV), a cue-based version of MRCP. Although their experiments were very systematic and concerned many aspects, they did not focus on self-paced experiments. In addition, effects of classifiers were also not mentioned in their work except for linear discrimination analysis (LDA). Furthermore, their results are limited to only offline usage due to the delay of the frequency filter. For other works, experiments were done under some constraints (e.g., a preset classifier, a preset frequency band, or a preset spatial filter). These situations could cause problems; for example, changing one factor may affect other factors as will be shown in our study. Also, the data were not acquired in a self-paced manner, nor the detection cannot be employed in real time [19], which makes the result unusable for movement intention detection problem.

In this paper, we make the first attempt to study effects and relationship among the 3 factors—frequency bands, spatial filtering techniques, and classifiers, specifically on EEG data acquired in a self-paced manner for movement intention detection. In detail, we recorded 19-channel EEG data from 9 subjects while performing a series of self-paced ankle dorsiflexions. After labeling and extracting the data, we employed frequency filters in the range of 0.01–5 Hz, where MRCP can be observed [25, 26]. Five well-known spatial filtering techniques and six classifiers were used. The five spatial filtering techniques are (1) no spatial filtering (NoF), (2) surface Laplacian (SL), (3) independent component analysis (ICA), (4) common spatial pattern analysis (CSP), and (5) principle component analysis (PCA). The six classifiers include (1) linear discrimination analysis (LDA), (2) support vector machine (SVM), (3) one-nearest neighbor utilizing Euclidean distance (1-NN-ED), (4) one-nearest neighbor utilizing dynamic time warping distance (1-NN-DTW), (5) shape-based template matching (TM), and (6) matched filter (MF). To emphasize, this study mainly focuses on finding relationship and effects of various factors and on determining good combinations among them. Our results provide valuable insights towards a development in an effective movement intention detection algorithm for asynchronous BCI rehabilitation systems.

Additionally, a novel shape-based template matching algorithm is added to our study to investigate a possibility of utilizing a shape of MRCP via time series mining techniques in BCI applications. Our study provides insight that can be used to help improve performance and efficiency of movement intention detection in asynchronous BCI systems.

Contributions and impact of this work can be summarized as follows:

- This work investigates relationship and effects of the three critical factors in asynchronous BCI systems for stroke rehabilitation. The results demonstrate that these factors are quite sensitive. Different combinations of the factors can substantially influence the system's performance.
- This work can be used as primitive guidelines to process self-paced EEG data in an asynchronous BCI system, which can assist the stroke rehabilitation.
- This work explores a possibility of utilizing shapes of time series signals to detect MRCP from self-paced EEG data using unconventional time series mining techniques.

## 2. Methodology

### 2.1. Subjects

Nine subjects (seven males and two females; age ranges from 22 to 26 years) participated in the experiment. None of the subjects had any known neurological disorders, nor had experiences with any BCI systems prior to the experiment. All subjects gave their signed informed consents for the experiments, and the experiment protocol was approved by the research ethics review committee for research involving human research participants, Health Science Group, Chulalongkorn University (COA No. 049/2018).

### 2.2. Experimental Protocol and Data Acquisition

**Figure 1:**
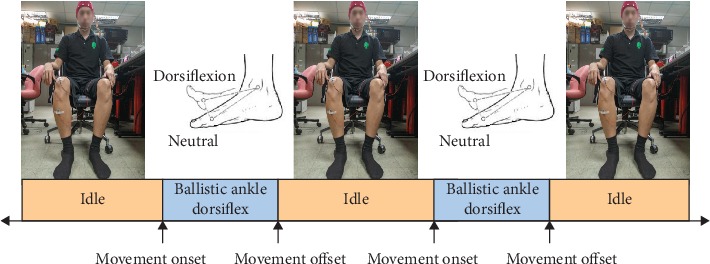
The experimental protocol shows the task of ballistic ankle dorsiflexion sequence followed by idle states. Idle states are as long as the user desires. These idle states normally take about 3 to 7 seconds.

At the beginning of the recording session, each participant was asked to sit on a chair in a comfortable position with both legs rested on the ground. After that, 19 channels of monopolar EEG were collected from each subject in the 10–20 system using Electro-Caps (Electro-Caps, Electro-Cap International Inc.) and a Nicolet w10-20HB amplifier (Natus Medical Inc.). The electrodes were located at Fp1, Fp2, F7, F3, Fz, F4, F8, T3, C3, Cz, C4, T4, T5, P3, Pz, P4, T6, O1, and O2. The ground electrode was the ground node of the cap that is in the middle among Fp1, Fp2, and Fz, and the reference was placed on the left earlobe. With the use of electrolyte gel, the impedance of all electrodes was calibrated to be less than the 5k-Ohm threshold before starting the data acquisition. One channel of surface electromyography (EMG) was also recorded by a Nicolet w10-20HB amplifier (with disposable electrodes) for the purpose of EEG signal labeling. EMG was recorded from bipolar derivation from the tibialis anterior (TA) muscle and on the bony surface of the knee of the dominant leg (right knees in all subjects). All of the EEG and EMG signals were sampled at 1024 Hz. EEG signals were filtered by a Butterworth band-pass filter with a frequency band of 0.01 to 30 Hz and a notch filter to filter out 49–51 Hz power-line distortion [27]. In the recording sessions, subjects were instructed to perform self-paced ballistic ankle dorsiflexions. The duration between consecutive trials was roughly 3 to 7 seconds. Roughly, there are about 280,000 data points per channel per session that need labeling. During the recording session to reduce artifacts, each subject was asked to stay relaxed, closing the eye lids, and not to move other body parts. The protocol is shown in Figure 1. Each subject took about six recording sessions with resting periods of two to ten minutes in between. During the recording sessions, videos were recorded for EMG signal synchronization. The recording was made in an environment similar to a hospital setting.

### 2.3. Data Labeling

After the EEG data acquisition, each period of the movement execution had to be labeled as a baseline for classification. To detect the onset and offset of each movement initiation and termination, respectively, EMG data were utilized in this process; EMG was visually inspected to discover movement onsets and offsets. Only trials whose EMG movement onset were at least 4 seconds apart from the previous offset were considered. Recorded raw data and their label are made publicly available as stated in the Data Availability section so that the experiment in this work can be reproduced.

### 2.4. Data Segmentation

The data were manually segmented (into 2-second sequences) for classification. For movement intention segments, we extracted a sequence starting before each actual movement and ending after the movement. In particular, each potential MRCP segment was extracted by considering a 2-second duration before each movement onset, and each non-MRCP segment was extracted by considering a 2-second duration right after each movement offset. After segmentation, there are 1,674 MRCP segments and 1,544 non-MRCP segments in total. All of the acquired segmented data were *z*-normalized. Further explanations and examples of segmented data can be downloaded via the link described in the Data Availability section.

### 2.5. Spatial Filtering Techniques

Electroencephalography (EEG) is an electrophysiological monitoring method to record electrical activities, i.e., voltage fluctuations within the neurons of the brain. However, these voltage fluctuations are so small (*μV*) that the EEG signal is easily contaminated by noise or even electric fields from other brain regions, resulting in a low signal-to-noise ratio of the acquired EEG. Many spatial filtering techniques have been introduced to accentuate localized EEG activities to maximize the signal-to-noise ratio. In this section, we describe some details of the widely used spatial filtering techniques that have been applied in our experiments to reduce noises or to enhance the quality of EEG signals.

#### 2.5.1. Surface Laplacian

SL is a technique used to reduce contamination in an electrode caused by other surrounding electrodes. SL is calculated by subtracting the weighted voltage of the surrounding electrodes from the working electrode. The weight is usually the distance from the electrode of interest to each of the surrounding electrodes. According to [28], the formula of SL is shown in the following equation:

(1) $$\begin{array}{c} V_{\text{surrogate}}=V_{\text{main}}-\underset{j\epsilon \text{NN}}{\sum }\frac{1/d_{j}}{\sum_{j\epsilon \text{NN}}\left(1/d_{j}\right)}V_{j}, \end{array}$$

where *V*_surrogate_ is the output voltage of SL, *V*_main_ is the voltage between the electrode of interest and a reference electrode, NN is a set of surrounding electrodes, *d*_*j*_ is the distance between *j* surrounding electrodes and the electrode of interest, and *V*_*j*_ is the voltage between the electrode *j* and the reference electrode. In our experiments, *d*_*j*_ was set to 1 for all *jϵ*NN and only nine channels, i.e., F3, Fz, F4, C3, Cz, C4, P3, Pz, and P4, were used as working electrodes as these electrodes have surrounding electrodes.

#### 2.5.2. Independent Component Analysis

ICA was invented to deal with problems similar to the cocktail-party problem. It can be used to separate a mixed signal into independent signals. In EEG domain, EEG signals are simultaneously acquired from many channels, thus a signal from one channel can be contaminated by the others. In this case, ICA is used to separate acquired signals into independent signals. According to [29], assume that we acquired *n* linear mixed signals from *n* channels *x*_1_,…, *x*_*n*_. Each signal *x*_*i*_ is originated from *n* independent components as illustrated in the following equation:

(2) $$\begin{array}{c} x_{1}=a_{1}s_{1}+a_{2}s_{2}+\cdots +a_{n}s_{n}. \end{array}$$

We can rewrite equation (2) in terms of vectors and matrix as shown in the following equation:

(3) $$\begin{array}{c} x=As, \end{array}$$

where *x* is a column vector form of *n* EEG channels, *s* is a column vector form of *n* independent components, and *A* is a matrix of size *n* × *n*. Instead of finding *x*, we want to find the inverse of *A*, denoted by *W*, to obtain the independent component *s*. A well-known algorithm for ICA is fastICA [29]. Instead of rejecting noisy components and reconstructing the original data as performed in [27], we used *W* to form independent components as new representations and feed them to the classifiers.

#### 2.5.3. Common Spatial Pattern

CSP is a method used for constructing a new representation from a high dimensional data to a lower dimensional data whose variance is maximized between two classes of data. In EEG domain, inputs of CSP are two classes of multichannel EEG data, i.e., MRCP and non-MRCP data. CSP then calculates a projection matrix that is used to project multichannel data into a low dimensional spatial subspace by a linear transformation [30]. The first and the last row of the projection matrix are most suitable for discriminating two different classes. They provide a transformation that maximizes a variance in one class and minimizes a variance in another. In this work, the first row of the projection matrix was utilized as a weight for multichannel EEG to make a new representation as performed in [16].

#### 2.5.4. Principle Component Analysis

PCA has been used as a dimensionality reduction technique for most data miners. However, PCA can also be used in data compression as it can bring out strong patterns from complex data while preserving variability. PCA projects the original data onto a new set of axes via its eigenvector, which is called principle component. Apart from the dimensionality reduction, PCA can also be used to decompose original data into a new set of decorrelated data by projecting the data onto orthogonal axes. Details of PCA, its principle, calculation, and application are well described in [31, 32]. In this work, we decomposed multichannel EEG data into independent components and then used them similarly to ICA.

### 2.6. Classifiers

#### 2.6.1. Linear Discriminant Analysis

LDA is one of the most widely used classification algorithms in BCI systems due to its simplicity and efficiency. It is based on the use of hyperplanes to separate different classes of data. A hyperplane can be determined by minimizing the intraclass variance and maximizing the distance between the means of two classes of data.

#### 2.6.2. Support Vector Machine

SVM [33] is based on an idea of hyperplane construction, which provides a maximum margin that can be used to separate the data. If the data are linearly separable in the input space, a hyperplane is constructed from the input data. Otherwise, a mapping of features to a higher dimensional space is done via a kernel function before a hyperplane construction. In this work, we used the *svm* function in MATLAB and performed optimization on all parameters using grid search with default settings.

#### 2.6.3. One-Nearest Neighbor

1-NN is a widely used classifier in a time series domain. A distance measure is used to measure similarity between two time series, in this case, two epochs of EEG data. A simple distance measure is Euclidean distance (ED), and a more complex one is dynamic time warping (DTW). The main difference between these two measures is that DTW has a nonlinear alignment ability, which makes DTW achieve higher accuracy than ED. The nonlinear alignment of DTW can be restricted to prevent unreasonable warping through the use of a global constraint, which in turn further increases the accuracy. In this work, 1-NN was utilized, while both ED and DTW were used as distance measures. For DTW, we used the Sakoe–Chiba band, which is one of the widely used global constraint, with *r*=10%. More information about DTW and the global constraints can be found in [34].

#### 2.6.4. Matched Filter

MF is a technique to extract a template, which is a known signal, in an unknown signal. MF can be calculated by convolving the unknown signal with a time-reversed template. In this work, an average of MRCP data was used to create a template.

#### 2.6.5. Template Matching

TM is similar to MF but different in template construction and similarity measurement. In time series mining, template construction can also be done through shape-based averaging. One of the most well-known shape-based averaging techniques for time series data is dynamic time warping barycenter averaging (DBA) [35], which had shown to outperform other existing averaging methods [35–37]. For similarity measurement, TM utilizes DTW as a similarity measure, while MF uses a convolution technique. In this work, we used DBA with a medoid of MRCP data as an initial template. The global constraint was set to 10% [38] for preventing unreasonable warp.

### 2.7. Experiment Setup

In the experiment, we started by filtering the acquired EEG data with a causal second-order Butterworth band-pass filter with frequency bands of [0.01-1], [1-2], [2-3], [3-4], and [4-5] Hz, followed by data segmentation. However, we set the order of Butterworth filter to 2^nd^ order and did not take other filter orders into account in this experiment due to an unstable problem of IIR filters as discussed in [20]. It is worth noting that there are infinitely many possible ways to extract frequency bands in the range of [0.01–5] Hz, where MRCP can be observed; thus, to make it feasible to extract only a prominent band and reject irrelevant ones, we decided to decompose the frequency into a bin of 1 Hz. Afterwards, MRCP and non-MRCP data were shuffled, and then stratified sampling was performed to create training and test sets with the ratio of 2/3 and 1/3, respectively. Overall numbers of MRCP and non-MRCP data for training set and test set of each subject are shown in Table 1. Then, five different spatial filtering techniques including no spatial filtering (NoF) were performed. For ICA and PCA, which require continuous data, MRCP and non-MRCP data of each training set were concatenated to form continuous data. To classify the MRCP data, six different classifying methods were used. To summarize, there were 5 frequency bands, 19 channels without spatial filter, 9 channels for SL, 19 components for ICA and PCA, 1 channel for CSP, and 6 classifying methods; thus, there were 5 × (19+9+19+19+1) × 6=2,010 combinations in total for each individual. By aiming to achieve high detection rate while providing low false alarm rate, we evaluated the results by measuring F1 scores. F1 scores are calculated by taking both precision and recall into account to maintain balance between them. Precision is a number of correctly predicted MRCP samples divided by a number of total samples predicted as MRCP. Recall is a number of correctly predicted MRCP samples divided by a number of total MRCP samples. F1 scores are calculated as shown in the following equation:

(4) $$\begin{array}{c} F1\text{ score}=\left(\frac{2}{{\text{recall}}^{-1}+{\text{precision}}^{-1}}\right). \end{array}$$

The best channel and component of each spatial technique and the threshold of the classifiers were selected from the best one that achieves the highest F1 score.

We employed three-way repeated measures analysis of variance (ANOVA) with Greenhouse–Geisser adjustment, which is used to adjust the degree of freedom, to investigate the effects of 3 independent variables, i.e., 5 spatial filters, 5 frequency bands, and 6 classifiers, on F1 scores. The total number of repeated measurements was 5 × 5 × 6=150. By employing ANOVA, we can investigate whether the variation of factors lead to significant difference of experimental results. In other words, the ANOVA can be used as an evidence to reject or accept the null hypothesis, which means the mean values of tested groups are the same. If the null hypothesis is rejected, the mean values of tested groups are considered different, and we can imply that the factors affect the results. On the other hand, if the null hypothesis is accepted, the mean values of tested groups can be considered similar, and the factors do not affect the results. The post hoc test used the Bonferroni adjustment to compensate for multiple comparisons. For the ANOVA test and the post hoc test, a significance level of *P* < 0.01 was adopted in this work. Due to the fact that our sample size is not considered very large, we decided to set the significance level of *P* < 0.01 instead of *P* < 0.1 or *P* < 0.05 as used in many previous works [8, 14, 16] for compensation and giving more confidence. The statistical analysis was performed by IBM SPSS Statistics 22.0.

## 3. Results

### 3.1. Three-Way Repeated Measures ANOVA

The output of three-way repeated measures ANOVA is shown in Table 2. There is a significant interaction effect between spatial filter and frequency, SF*∗*Freq · (*F*(3.659, 29.275)=10.811, *P* < 0.01). None of other interaction effects are significant. This means the effect of frequency and the effect of spatial filter are dependent in some circumstances, which will also be explored in this work. There is also a significant effect of the frequency, Freq(*F*(2.167, 17.339)=21.384, *P* < 0.01), and the classifier, Classifier(*F*(1.24, 9.921)=13.102, *P*=0.003). No other significant effects are found.

**Figure 2:**
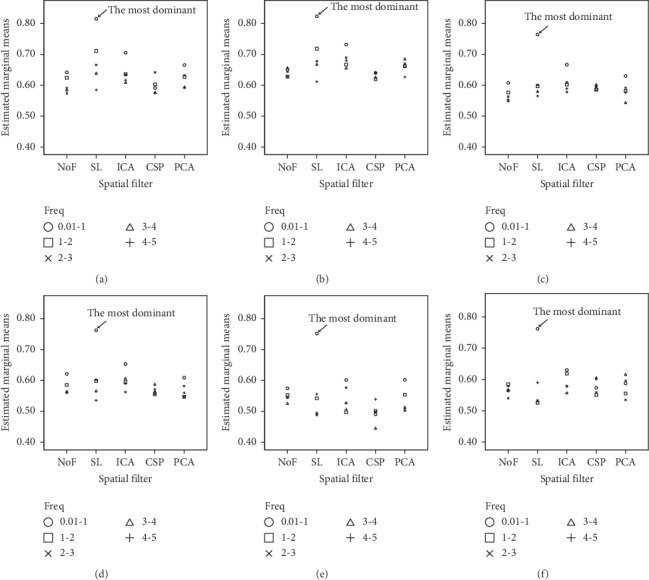
Multiple comparison results of the three-way interactions among different spatial filter, frequency, and classifier. The best combination is when classifier = SVM, spatial filter = SL, and frequency = [0.01–1] Hz. (a) Estimated marginal means of F1 score with classifier = LDA. (b) Estimated marginal means of F1 score with classifier = SVM. (c) Estimated marginal means of F1 score with classifier = 1-NN-ED. (d) Estimated marginal means of F1 score with classifier = 1-NN-DTW. (e) Estimated marginal means of F1 score with classifier = MF. (f) Estimated marginal means of F1 score with classifier = TM.

To visualize the three-way interaction, we illustrate multiple comparison graphs of the three-way interaction in Figure 2. The highest estimated marginal mean of F1 scores can be found by utilizing EEG data in frequency [0.01-1] Hz with SL, regardless of the choice of classifier. For the classifier, also shown in Figure 2, LDA and SVM provide comparable F1 scores; the rest provide relatively lower F1 scores than LDA and SVM, while providing comparable F1 scores to each other.

The results in Figure 2 also show that these factors are quite sensitive. Choosing different combinations of the factors can substantially affect the F1 scores. The scores could range from as low as 44.4% with the choice of [3-4] Hz frequency + CSP filter + MF classifier to as high as 82.3% with the choice of [0.01-1] Hz frequency + SL spatial filter + SVM classifier.

**Figure 3:**
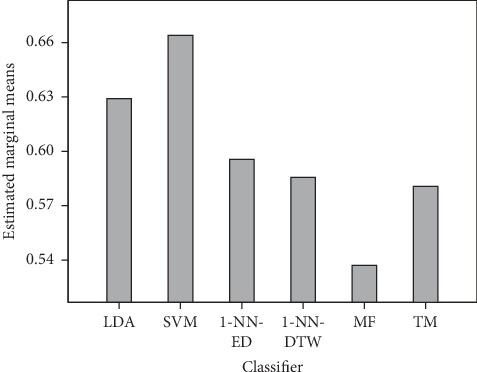
Comparison of the estimated marginal means of F1 scores in various classifiers, showing SVM as the dominant classifier.

After a post hoc test of the classifier, we found that SVM is the most prominent classifier, being an independent factor without any intervention from other factors. The estimated marginal mean of SVM is 66.4% with a significantly higher F1 score than the others including LDA, as shown in Figure 3.

The estimated marginal mean difference between SVM and LDA is about 4%. Moreover, the multiple comparison results of the classifiers revealed that LDA is the second most prominent classifier providing high F1 scores with a significant difference from the other classifiers. 1-NN-ED, 1-NN-DTW, and TM provide comparable F1 scores among each other, i.e., 59.6%, 58.6%, and 58.1%, respectively. MF is the worst classifier providing the lowest F1 score of 53.7%.

From the three-way repeated measures ANOVA test, the result tells us that frequency has a significant impact while spatial filter has no significant impact. However, since frequency and spatial filter are highly correlated, we need to conduct one-way repeated measures ANOVA to investigate simple effects of spatial filter at every single level of frequency and also simple effects of frequency at every single level of spatial filter.

### 3.2. One-Way Repeated Measures ANOVA

The results of simple effects of spatial filter at every single level of frequency are shown in Table 3. Interestingly, a significant simple effect of spatial filter can be found only in the frequency band of [0.01-1] Hz, SF at [0.01-1] Hz (*F*(3.463, 183.548)=76.338, *P* < 0.01). No other significant effect of spatial filter can be found when using other levels of frequency. This result means that a spatial filter can be arbitrarily chosen in any frequency band except in [0.01-1] Hz, which has to be precisely chosen to provide valuable results.

**Figure 4:**
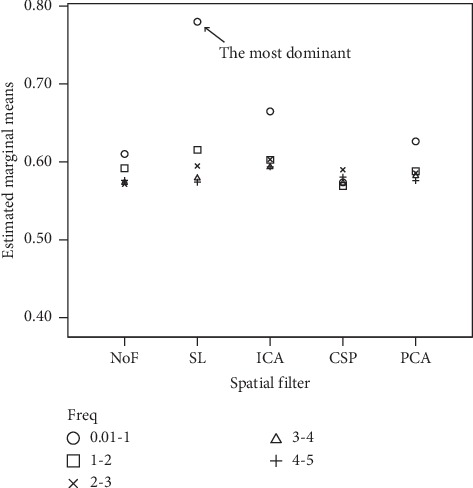
Comparison of the estimated marginal means of F1 scores in various frequency bands on different spatial filters. SL spatial filter at [0.01-1] Hz frequency clearly provides the best result.

To visualize the effects, we plot a multiple comparison graph for the simple effects of spatial filter at each level of frequency in Figure 4.

In particular, NoF, SL, ICA, CSP, and PCA for EEG data in [0.01-1] Hz frequency provide estimated marginal means of F1 scores of 61%, 78%, 66.5%, 57.4%, and 62.6%, respectively.

After post hoc tests, there is a significant difference when comparing NoF with SL and ICA, but there is no significant difference between CSP and PCA. SL provides the highest F1 score, and it is significantly different from the rest when the frequency is fixed to [0.01-1] Hz. ICA is the second best choice of spatial filter in this bandwidth. It provides the second highest F1 score with a significant difference from other spatial filters. CSP and PCA are also significantly different from each other but they are insignificantly different from NoF.

Next, we provide a further exploration to investigate simple effects of frequency at each type of spatial filters. We therefore conducted one-way repeated measures ANOVA by fixing spatial filter types and adjusting the frequency bandwidth. The output is shown in Table 4. Frequency gives significant effects on NoF, SL, ICA, and PCA: Freq on NoF (*F*(3.303, 175.053)=5.536, *P*=0.001), Freq on SL (*F*(3.167, 167.856)=70.402, *P* < 0.01), Freq on ICA (*F*(3.085, 163.490)=10.265, *P* < 0.01), and Freq on PCA(*F*(3.433, 181.937)=6.094, *P* < 0.01), respectively. No significant effect of frequency is found on CSP.

After post hoc tests, pairwise comparison of estimated marginal mean of F1 scores reveals that for NoF, only one pair of [0.01-1] and [2-3] Hz frequency bands does have significantly different scores; the rest of the frequency bands are found not to be significantly different from each other. For SL and ICA, the [0.01-1] Hz frequency band provides a prominent estimated marginal mean of F1 score, which made this bandwidth significantly different to all others, while the rest are insignificantly different. For PCA, there are only two pairs of frequency bands that provide significant difference of estimated marginal mean of F1 score; the first pair is [0.01-1] and [3-4] Hz; the second is [0.01-1] and [4-5] Hz. These results are illustrated in Figure 4.

### 3.3. Localization of MRCP Detection Performance

**Figure 5:**
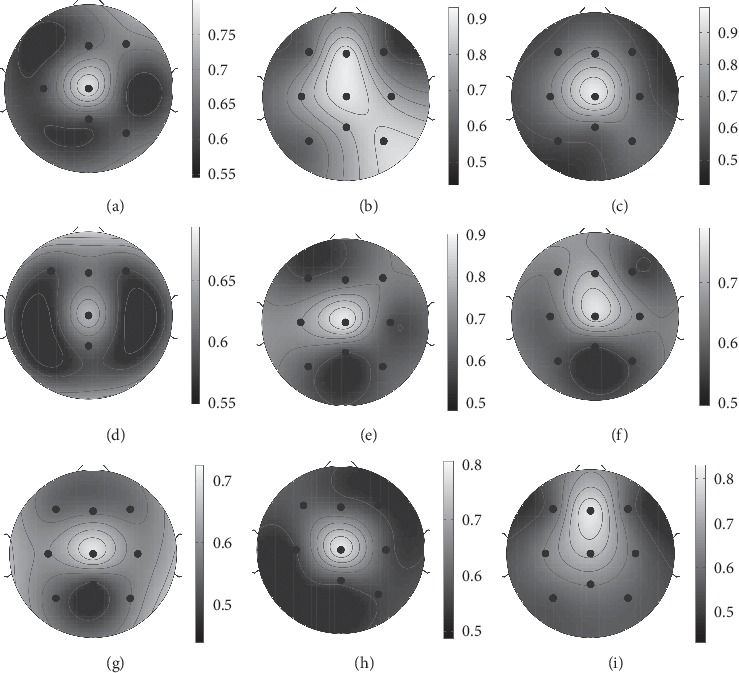
The topoplot demonstrates the F1 score of each channel for every subject when frequency was set with [0.01-1] Hz using SL spatial filter. The channels with higher discriminant power appear brighter. (a) Subject 1. (b) Subject 2. (c) Subject 3. (d) Subject 4. (e) Subject 5. (f) Subject 6. (g) Subject 7. (h) Subject 8. (i) Subject 9.

Furthermore, to investigate MRCP localization diagnosis accuracy, we provide a topoplot of F1 score in Figure 5 corresponding to a surrogate channel of SL at frequency [0.01-1] Hz, which is the best combination among these factors for each subject. The channel that provides prominent F1 scores for each subject is Cz in most subjects except in subject 2 where both Cz and Fz are prominent and in subject 9 where Fz provides higher F1 score than Cz.

The results in Figure 2 also show that these factors are quite sensitive. Choosing different combinations of the factors can substantially affect the F1 scores. The scores could range from as low as 44.4% with the choice of [3-4] Hz frequency + CSP filter + MF classifier to as high as 82.3% with the choice of [0.01-1] Hz frequency + SL spatial filter + SVM classifier.

After a post hoc test of the classifier, we found that SVM is the most prominent classifier, being an independent factor without any intervention from other factors. The estimated marginal mean of SVM is 66.4% with a significantly higher F1 score than the others including LDA, as shown in Figure 3.

## 4. Discussion

This study aimed to investigate factors that affect performance of MRCP detection in asynchronous BCI, which can facilitate stroke rehabilitation systems. To tackle this problem, we carried out a systematic analysis on effects of three prevalent factors including 5 different frequency bands of the EEG data, 5 spatial filters, and 6 classification algorithms. To focus on both high detection rate and quality of the detection, we analyzed these factors using F1 scores. Series of ANOVA tests were conducted to investigate relationship and effects among these factors based on F1 scores.

Interestingly, the results show that there is no significant relationship in three-way interaction. However, in two-way interaction, there is a significant interactive effect between frequency and spatial filter. This means that the effect of frequency and the effect of spatial filter are dependent in some circumstances. When EEG data were filtered to the frequency band of [0.01-1] Hz, applying different spatial filters will provide significantly different F1 scores. Nonetheless, results were not significantly different regarding different spatial filters in other frequency bands. In other words, the spatial filter only has to be carefully and precisely chosen when used with EEG data in [0.01-1] Hz range. More specifically, the experiment results show that utilizing frequency in [0.01-1] Hz with SL spatial filter provided best F1 scores. This can be interpreted as the discriminant feature of MRCP may be discovered only in frequency band of [0.01-1] Hz and not all types of spatial filter can reveal this discriminant feature. However, we also respect the fact that different experiment protocols and different methods in acquiring EEG data may affect the setting in the preprocessing step; our experiment intends to reveal an influence, effect, and interaction among these factors more than to discover the best setting for the preprocessing step under some preset factors as done in previous works [20, 21, 24]. By pointing out that not all the settings can enhance the accuracy, we suggest researchers who conduct research in this area select these factors in the preprocessing method with extra care.

The classifying method does not have any interaction effects with other factors, meaning that an appropriate classifier will provide good F1 scores regardless of the frequency bands and spatial filters used. The most prominent classifier for MRCP detection is SVM, while LDA is the second best. 1-NN-ED, 1-NN-DTW, and TM provide similar results, while MF provides the worst F1 scores. These results are according to the fact that EEG data are nonstationary signals; thus, a classification algorithm which is a stable learning algorithm like SVM and LDA can provide superior results than 1-NN-ED and 1-NN-DTW [26]. Although MF and TM are members of stable learning algorithm, neither MF nor TM provides comparable F1 score to SVM and LDA. We notice that compressing nonstationary signals into one template as done in MF and TM is not a good solution for dealing with EEG data. However, TM provides comparable F1 scores to 1-NN-ED and 1-NN-DTW, but not MF. This is resulted from the shape-based averaging that tries to average the data based on its shape.

Moreover, the distance measures do not significantly affect the results when EEG data are used in time domain as 1-NN-ED and 1-NN-DTW provide similar results. If memory space is taken into account, TM will be superior to 1-NN-ED and 1-NN-DTW. These three classifiers provide comparable F1 scores; however, 1-NN-ED and 1-NN-DTW use a lazy learning method that keeps all training instances in memory to make a decision. Instead of keeping all available training instances, MF and TM create templates of groups of training data. Thus, it reduces memory usage and decision time. For MF and TM that needs template constructions, TM is a better choice than MF because TM employs a shape-based averaging technique, whereas MF uses a simple mean averaging method. Furthermore, utilizing TM could provide comparable F1 scores with 1-NN-DTW, which means that the shape-based averaging method such as DBA can reserve important information, while the number of kept instances in memory is minimized.

## 5. Conclusion

We performed a comprehensive statistical analysis on performance of different frequency bands, spatial filters, and classifying methods to reveal effects and relationship among these factors. The performance was analyzed based on F1 scores. The results showed that the classifier is an independent factor, whereas the frequency and the spatial filter are dependent factors. Thus, the frequency and the spatial filter have to be considered simultaneously. These results can be used as guidelines for research in MRCP detection, especially on lower limb movement. For time series miners who are interested in developing an MRCP detection algorithm, either a more sophisticated data representation or projection for MRCP is required to provide superior results.

## Figures and Tables

**Table 1:** Number of samples in MRCP class vs. non-MRCP class of all participants.

	Training set		Test set	
Subject	MRCP	Non-MRCP	MRCP	Non-MRCP
1	122	108	62	55
2	120	113	61	57
3	124	106	63	54
4	118	111	60	57
5	116	103	59	53
6	99	90	51	46
7	142	135	72	69
8	140	135	71	69
9	129	121	65	62

**Table 2:** Results of the three-way repeated measures ANOVA test of F1 scores.

Source	Type III sum of squares	Corrected degree of freedom	Mean square	*F* value	*P* value	Partial eta squared
SF	0.478	1.273	0.375	7.163	0.018	0.472
Error (SF)	0.534	10.185	0.052			

Freq	0.958	2.167	0.442	21.384	**0.000**	0.728
Error (freq)	0.359	17.339	0.021			

Classifier	2.135	1.24	1.721	13.102	**0.003**	0.621
Error (classifier)	1.303	9.921	0.131			

SF ∗ Freq	0.999	3.659	0.273	10.811	**0.000**	0.575
Error (SF ∗ Freq)	0.739	29.275	0.025			

SF ∗ Classifier	0.172	4.088	0.042	1.661	0.182	0.172
Error (SF ∗ Classifier)	0.827	32.705	0.025			

Freq ∗ Classifier	0.135	4.859	0.028	1.676	0.165	0.173
Error (Freq ∗ Classifier)	0.644	38.870	0.017			

SF ∗ Freq ∗ Classifier	0.397	6.268	0.063	1.299	0.274	0.14
Error (SF∗Freq ∗ Classifier)	2.445	50.143	0.049			

*P* value less than 0.01 is shown in bold, showing significant effects of the parameter to F1 scores. Note that there is a significant interaction effect between spatial filter and frequency; further analysis is needed for these two parameters.

**Table 3:** Simple effect results of spatial filter with each frequency band from the one-way repeated measures ANOVA test of F1 scores.

Source	Type III sum of squares	Corrected degree of freedom	Mean square	*F* value	*P* value	Partial eta squared
SF at [0.01-1] Hz	1.354	3.463	0.391	76.338	**0.000**	0.590
Error (SF at [0.01-1] Hz)	0.940	183.548	0.005			

SF at [1-2] Hz	0.064	3.551	0.018	2.768	0.034	0.050
Error (SF at [1-2] Hz)	1.232	188.184	0.007			

SF at [2-3] Hz	0.030	3.212	0.009	1.397	0.244	0.026
Error (SF at [2-3] Hz)	1.132	170.222	0.007			

SF at [3-4] Hz	0.015	2.809	0.005	0.755	0.513	0.014
Error (SF at [3-4] Hz)	1.082	148.867	0.007			

SF at [4-5] Hz	0.014	3.318	0.004	0.990	0.404	0.018
Error (SF at [4-5] Hz)	0.728	175.831	0.004			

The only significant simple effect of the spatial filter can be found only in the frequency band of [0.01-1] Hz, where the *P* value is less than 0.01.

**Table 4:** Simple effects of frequency with each type of spatial filter from the one-way repeated measures ANOVA test of F1 scores.

Source	Type III sum of squares	Corrected degree of freedom	Mean square	*F* value	*P* value	Partial eta squared
Freq on NoF	0.058	3.303	0.018	5.536	**0.001**	0.095
Error (freq on NoF)	0.557	175.053	0.003			

Freq on SL	1.602	3.167	0.506	70.402	**0.000**	0.571
Error (freq on SL)	1.206	167.856	0.007			

Freq on ICA	0.196	3.085	0.064	10.265	**0.000**	0.162
Error (freq on ICA)	1.014	163.490	0.006			

Freq on CSP	0.015	2.743	0.005	0.643	0.575	0.012
Error (freq on CSP)	1.196	145.357	0.008			

Freq on PCA	0.086	3.433	0.025	6.094	**0.000**	0.103
Error (freq on PCA)	0.746	181.937	0.004			

There are significant simple effects of frequency band on all types of spatial filter but not CSP, where *P* value is more than 0.01.

## Data Availability

The BCI data used to support the findings of this study are available at http://www.cu-timeseries.com or from the corresponding author upon request.
